# Supplementary material for: Causal Relationship between Aging and Anorexia Nervosa: A White-Matter-Microstructure-Mediated Mendelian Randomization Analysis
Source: Biomedicines. 2024 Aug 16;12(8):1874. doi: 10.3390/biomedicines12081874 (PMC11351342; doi:10.3390/biomedicines12081874)
Supplement: Supplementary file 1 [file biomedicines-12-01874-s001.zip › Supplementary Figure/Figure S4/Singe SNP plot of MR analysis for TL to ubm-b-1677.pdf]

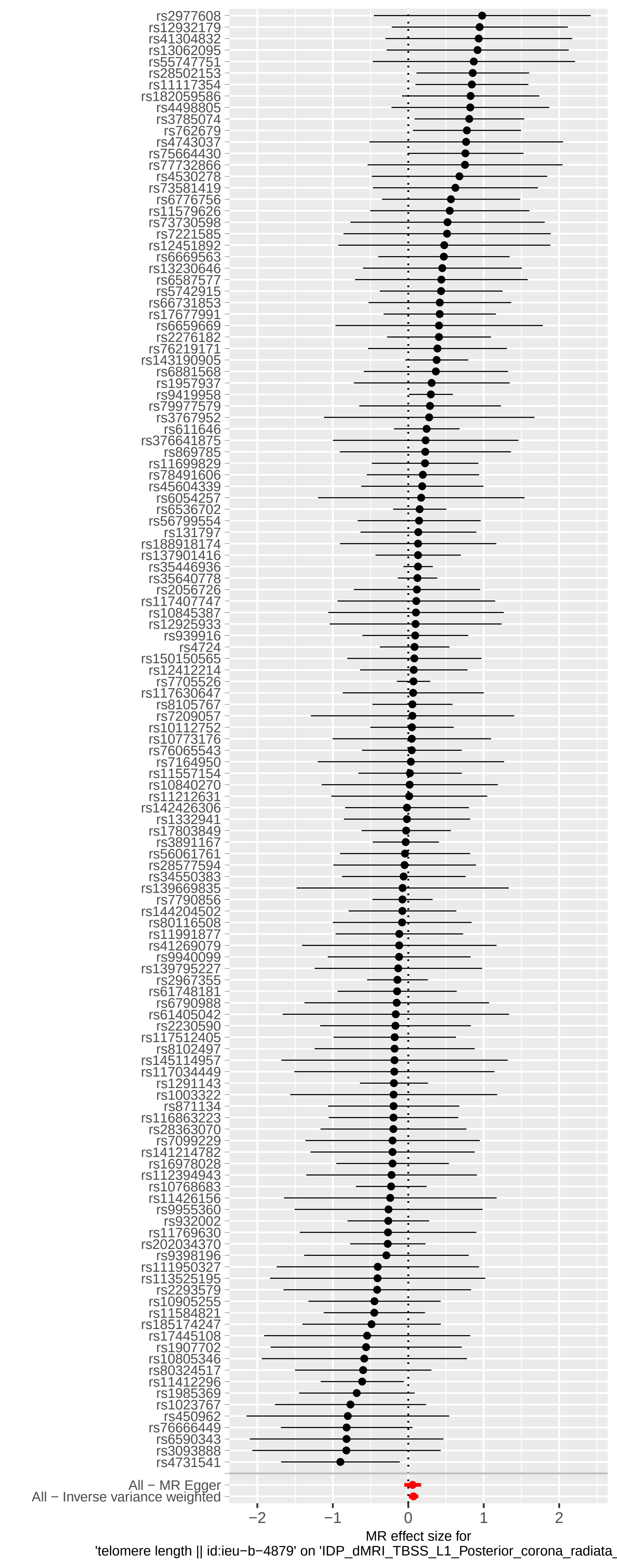

rs2977608
rs12932179
rs41304832
rs13062095
rs55747751
rs28502153
rs11117354
rs182059586
rs4498805
rs3785074
rs762679
rs4743037
rs75664430
rs77732866
rs4530278
rs73581419
rs6776756
rs11579626
rs73730598
rs7221585
rs12451892
rs6669563
rs13230646
rs6587577
rs5742915
rs66731853
rs17677991
rs6659669
rs2276182
rs76219171
rs143190905
rs6881568
rs1957937
rs9419958
rs79977579
rs3767952
rs611646
rs376641875
rs869785
rs11699829
rs78491606
rs45604339
rs6054257
rs6536702
rs56799554
rs131797
rs188918174
rs137901416
rs35446936
rs35640778
rs2056726
rs117407747
rs10845387
rs12925933
rs939916
rs4724
rs150150565
rs12412214
rs7705526
rs117630647
rs8105767
rs7209057
rs10112752
rs10773176
rs76065543
rs7164950
rs11557154
rs10840270
rs11212631
rs142426306
rs1332941
rs17803849
rs3891167
rs56061761
rs28577594
rs34550383
rs139669835
rs7790856
rs144204502
rs80116508
rs11991877
rs41269079
rs9940099
rs139795227
rs2967355
rs61748181
rs6790988
rs61405042
rs2230590
rs117512405
rs8102497
rs145114957
rs117034449
rs1291143
rs1003322
rs871134
rs116863223
rs28363070
rs7099229
rs141214782
rs16978028
rs112394943
rs10768683
rs11426156
rs9955360
rs932002
rs11769630
rs202034370
rs9398196
rs111950327
rs113525195
rs2293579
rs10905255
rs11584821
rs185174247
rs17445108
rs1907702
rs10805346
rs80324517
rs11412296
rs1985369
rs1023767
rs450962
rs76666449
rs6590343
rs3093888
rs4731541
All − MR Egger
All − Inverse variance weighted
−2
−1
0
1
2
MR effect size for
'telomere length || id:ieu−b−4879' on 'IDP_dMRI_TBSS_L1_Posterior_corona_radiata_
